# Cyclosporine A protects podocytes by regulating WAVE1 phosphorylation

**DOI:** 10.1038/srep17694

**Published:** 2015-12-04

**Authors:** Xuejuan Li, Fangrui Ding, Suxia Wang, Baihong Li, Jie Ding

**Affiliations:** 1 Department of Pediatrics, Peking University First Hospital, Beijing 100034, China; 2 Electron Microscopy Laboratory, Peking University First Hospital, Beijing 100034, China

## Abstract

Accumulating evidence suggests that podocytes are direct targets of many classic antiproteinuric drugs. The immunosuppressive drug cyclosporine A (CsA), which is a calcineurin inhibitor, is used to treat proteinuric kidney diseases. One novel mechanism by which CsA reduces proteinuria is by directly stabilizing the podocyte cytoskeleton. Previous studies showed that calcineurin can directly regulate WAVE1 within mouse striatal slices. In this study, WAVE1 was expressed in podocytes and was localized in the podocyte cell bodies and foot processes (FPs). WAVE1 expression increased in both *in vivo* and *in vitro* models of puromycin aminonucleoside (PAN)-induced podocyte injury. CsA restored WAVE1 expression and also partially rescued the disordered F-actin arrangement after PAN injury. Co-immunoprecipitation assays showed that calcineurin directly interacted with WAVE1 and regulated WAVE1 phosphorylation in podocytes. Synaptopodin is a well-characterized target of CsA. WAVE1 overexpression and synaptopodin knockdown experiments directly demonstrated that WAVE1 expression is not dependent on synaptopodin expression, and vice versa. Overexpression of WAVE1 using a WAVE1 plasmid disrupted F-actin structure and promoted podocyte migration compared with the empty vector group. Therefore, WAVE1 may be a novel molecular target for the maintenance of podocyte FPs and for antiproteinuric treatment in the future.

Proteinuria is one of the most common manifestations of kidney disease, and it is a major risk factor for the progression of kidney disease to end-stage renal failure^1^. In recent years, many reports have shown that altered podocyte actin cytoskeletal structure is a common event that leads to podocyte foot process (FP) effacement and proteinuria^2^,^3^,^4^,^5^,^6^,^7^,^8^. It is now widely accepted that the podocyte is a direct target of many classic antiproteinuric drugs. Of these, cyclosporine A (CsA) is one of the most widely utilized drugs to treat proteinuria in renal diseases^9^,^10^. Although the traditional mechanism of CsA-mediated immunosuppression involves the inhibition of nuclear factor of activated T cells (NFAT) signalling in T cells^11^, the calcineurin inhibitor CsA reduces proteinuria by directly stabilizing the podocyte cytoskeletal structure. CsA has been reported to block the calcineurin-mediated dephosphorylation of synaptopodin^12^, a podocyte-specific and actin-regulated protein, and protect synaptopodin from cathepsin L-mediated degradation, which in turns stabilizes the podocyte actin cytoskeleton and cofilin1^13^. However, it is unclear whether there are other targets of CsA.

In 2010, Ceglia *et al.*^14^ reported that calcineurin regulates Wiskott-Aldrich syndrome protein (WASP)-family protein 1 (WAVE1) by dephosphorylating serine residues that are phosphorylated by different factors in mouse striatal slices. Although the precise mechanism by which calcineurin regulates this pathway is unknown, this previous report demonstrated that WAVE1 might be a novel substrate of calcineurin in mouse striatal tissue. WAVE1 is a member of the WASP family, a key regulator of Arp (actin-related protein) 2/3 complex-mediated actin polymerization, and an important actin nucleator^15^,^16^,^17^. WAVE1 is expressed most abundantly in murine brain tissue and at low levels in other tissues, including the heart, liver, lung, kidney, pancreas, and peripheral blood^18^. In 1998, Miki *et al.*^19^ demonstrated that WAVE1 regulates actin reorganization downstream of the Rho family GTPase Rac. Soon after, they also reported that WAVE is a phosphoprotein whose phosphorylation increased in Swiss 3T3 cells stimulated with platelet-derived growth factor, which activated mitogen-activated protein (MAP) kinase signaling^20^.

However, whether WAVE1 is a novel substrate in the regulation of the podocyte cytoskeleton is unknown, as is its expression in podocytes. Here, we report the expression and distribution of WAVE1 in kidney glomerular podocytes. We demonstrated that WAVE1 might be involved in podocyte injury and might regulate the stabilization of the podocyte actin cytoskeleton. In particular, we showed that calcineurin directly interacted with WAVE1 and regulated WAVE1 phosphorylation in podocytes.

## Results

### WAVE1 expression in glomerular podocytes

**Figure 1:**
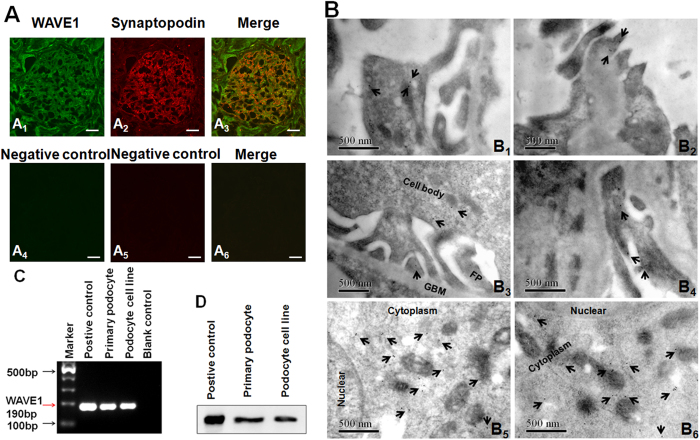
WAVE1 is expressed in glomerular podocytes. (**A**) Double-immunolabelling of human normal kidney sections revealed a WAVE1 glomerular signal that partially overlaps with the podocyte marker synaptopodin(A_1–3_), Images A_4–6_ are the negative controls by without primary antibodies. Scale bar = 20 μm. (**B**) Immunoelectron microscopy revealed that WAVE1 localizes to the glomerular podocyte cell body and to the apical side of foot processes (FPs) in rat normal kidney sections (B_1–4_), primary cultured mouse podocytes (B_5_) and human glioma cells (the positive control) (B_6_). In the cultured cells, WAVE1 was mainly distributed in the cytoplasm. Scale bar = 500 nm. (**C**) Conventional PCR demonstrated WAVE1 expression in primary cultured podocytes. (**D**) Western blotting revealed specific WAVE1 protein expression in primary cultured podocytes. Mouse cerebral cortex tissue was used as the positive control. n = 3.

WAVE1 was mainly localized in the glomerular capillary walls, where it partially overlapped with staining for synaptopodin (red fluorescence) via immunofluorescence in human normal kidney sections (Fig. 1A). Immunoelectron microscopy (IEM) was performed on normal rat kidney sections with an antibody against WAVE1 to resolve the subcellular localization of WAVE1. Most of the positive immunogold particles were observed in the podocyte cell body and the apical side of FPs (Fig. 1B). Labelling was also detected in endothelial cells and to a minor extent in mesangial cells (data not shown). We used conventional PCR and Western blotting to further confirm WAVE1 mRNA (Fig. 1C) and protein (Fig. 1D) expression in primary cultured podocytes. Mouse cerebral cortex tissue served as the positive control.

### WAVE1 expression increased after PAN-induced podocyte injury

**Figure 2:**
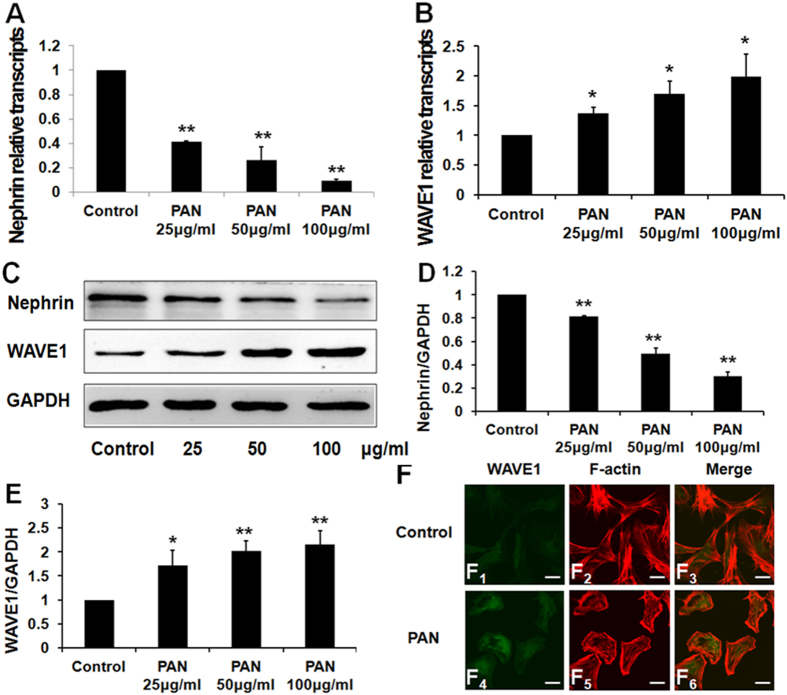
WAVE1 expression increases in podocytes after 24-h PAN treatment. (**A**,**B**) Nephrin and WAVE1 mRNA expression levels were evaluated by quantitative real-time PCR. (**C**) Nephrin and WAVE1 protein expression levels were measured by Western blotting. (**D**,**E**) Protein levels were quantified and normalized to GAPDH expression. (**F**) Double-immunolabelling of WAVE1 and F-actin in primary cultured podocytes. Scale bar = 20 μm. WAVE1 is labelled in green, and F-actin is labelled in red. The data are presented as the mean ± SD. n = 3. *P < 0.05, **P < 0.01.

Podocytes were treated with different doses of PAN. WAVE1 mRNA and protein expression levels were detected by quantitative real-time PCR and Western blotting, respectively. WAVE1 expression significantly increased after PAN treatment (Fig. 2A–E). Immunofluorescence staining showed that WAVE1 was mainly localized in the cytoplasm in normal primary mouse podocytes, whereas the fluorescence intensity increased markedly in the cytoplasm and moved to the periphery of the nucleus after treatment with PAN for 24 h (Fig. 2F_1_,F_4_). Our data therefore suggested that WAVE1 was involved in podocyte injury.

### Effects of CsA on podocyte WAVE1 expression in PAN-induced rat nephropathy

**Figure 3:**
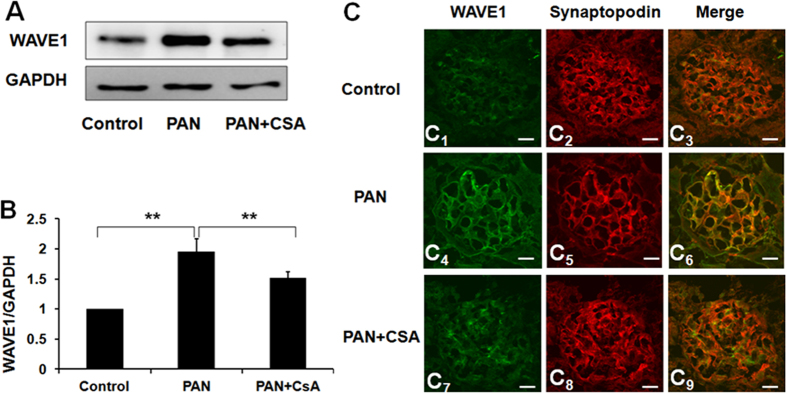
Effects of CsA on podocyte WAVE1 expression in PAN-induced rat nephropathy. (**A**) Western blot analysis of WAVE1 in isolated glomeruli. (**B**) WAVE1 expression was quantified and normalized to GAPDH expression. (**C**) Immunofluorescent staining of WAVE1 and synaptopodin in rats. Scale bar = 20 μm. WAVE1 is labelled in green, and synaptopodin is labelled red. The data are presented as the mean ± SD. n = 5. **P < 0.01.

WAVE1 expression significantly increased on the 10th day after PAN injection compared with the control group. However, CsA treatment restored WAVE1 expression to control levels (Fig. 3A,B). WAVE1 is a key regulator of Arp 2/3 complex-mediated actin polymerization. We assessed the expression and distribution of WAVE1 by immunofluorescence. WAVE1 expression in glomerular capillary walls increased significantly by day 10 after PAN injection compared with controls, and CsA treatment significantly restored WAVE1 expression. However, no significant changes in protein distribution were observed (Fig. 3C_1_,C_4_,C_7_). Synaptopodin, an actin binding protein, is a podocyte-specific marker. Double-labelling assays revealed that WAVE1 partly colocalized with synaptopodin (Fig. 3C_3_,C_6_,C_9_). These observations indicated that WAVE1 was involved in PAN-induced podocyte injury and in the beneficial effect of CsA *in vivo*. We next explored whether CsA could restore podocyte WAVE1 expression in an *in vitro* model of PAN-induced podocyte injury.

### Effects of CsA on podocyte WAVE1 expression in PAN-induced podocyte injury *in vitro*

**Figure 4:**
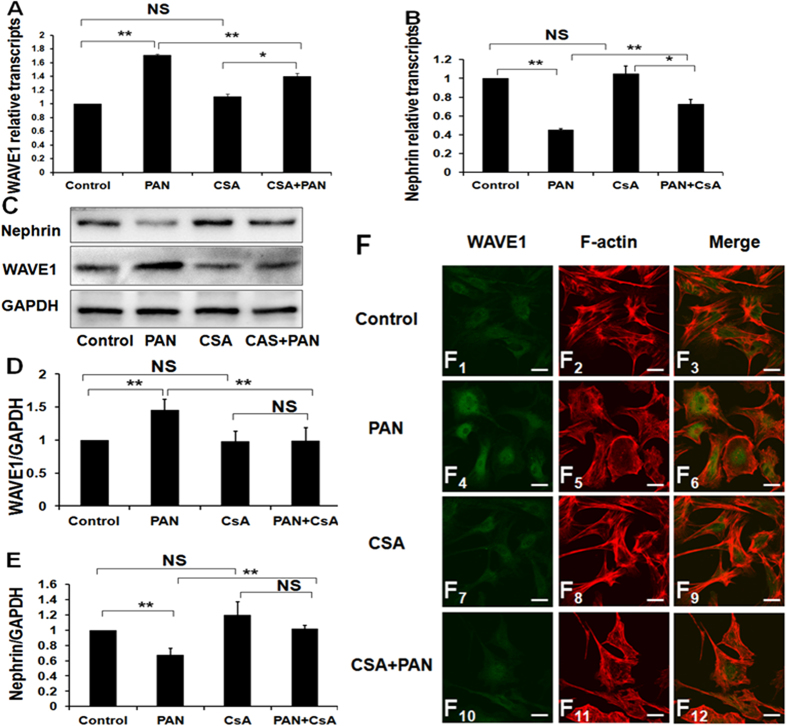
Effects of CsA on podocyte WAVE1 expression in an *in vitro* model of PAN-induced podocyte injury. (**A**,**B**) Nephrin and WAVE1 mRNA expression levels were evaluated by quantitative real-time PCR in podocytes. (**C**) Nephrin and WAVE1 protein expression levels were determined by Western blotting. (**D**,**E**) Protein expression was quantified and normalized to GAPDH expression. (**F**) Double-immunolabelling of WAVE1 and F-actin in primary cultured podocytes. Scale bar = 20 μm. WAVE1 is labelled in green, and F-actin is labelled in red. The data are presented as the mean ± SD. n = 3. *P < 0.05, **P < 0.01, NS, not significant.

Quantitative real-time PCR and Western blotting revealed that WAVE1 expression increased significantly after treatment with PAN for 24 h, whereas CsA treatment significantly decreased WAVE1 expression (Fig. 4A,C,D). In addition, we used nephrin as a marker of podocyte injury. Nephrin expression decreased significantly after treatment with PAN for 24 h, but CsA treatment restored nephrin expression (Fig. 4B,C,E).

CsA treatment partially restored WAVE1 expression, as evidenced by immunofluorescence staining (Fig. 4F_4_,F_10_). In normal podocytes, F-actin forms highly ordered, parallel, contractile actin filament bundles. After PAN injury, the cytoplasm was filled with rearranged, short, branched, and disorganized actin filaments. CsA treatment partially recovered the F-actin arrangement (Fig. 4F11). The merged images showed that WAVE1 partly colocalized with F-actin (Fig. 4F3,F6,F12).

### Protective role of CsA in PAN-induced rat nephropathy

**Figure 5:**
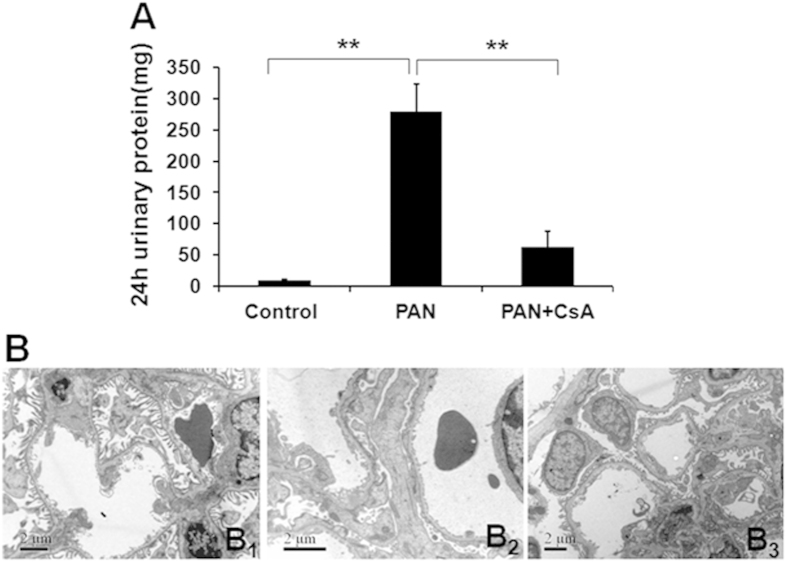
Twenty-four-hour urinary protein and ultrastructural changes in podocyte FPs in a rat model. (**A**) Compared with the control groups, proteinuria significantly increased 10 days after PAN injection. The proteinuria level decreased significantly with CsA treatment. The data are presented as the mean ± SD. n = 5. **P < 0.01. (B_1_) The FPs were long and thin in the control group. (B_2_) Ten days after PAN injection, FPs showed widespread effacement and were diffuse. (B_3_) Typical FPs were observed after treatment with CsA. Scale bar = 2 μm.

Proteinuria levels increased sharply in PAN-induced rats versus controls by day 10 (278.6 ± 44.3 mg/24 h versus 9.9 ± 0.8 mg/24 h, P < 0.01). CsA treatment significantly attenuated proteinuria (94.3 ±52.9 mg/24 h versus 278.6 ±  44.3 mg/24 h, P < 0.01) (Fig. 5A). The FPs of normal rats were long and thin (Fig. 5B1). Ten days after PAN injection, podocyte FPs showed diffuse effacement. The FP structures were partially recovered in the CsA-treatment group compared with the PAN group (Fig. 5B_2_,B_3_).

### Calcineurin directly interacted with WAVE1 and regulated WAVE1 phosphorylation in podocytes

**Figure 6:**
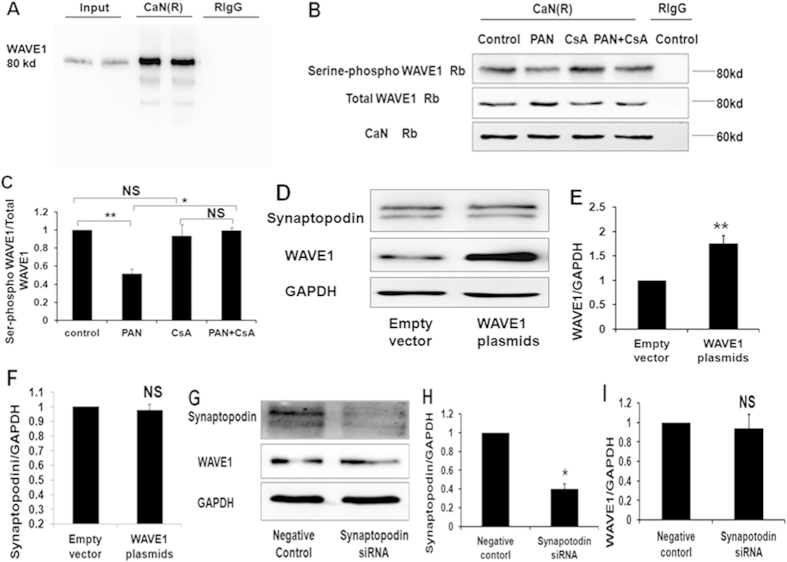
The interaction between WAVE1 and calcineurin and WAVE1 phosphorylation in PAN-stimulated podocytes. (**A**) Co-immunoprecipitation analysis of the interaction between WAVE1 and calcineurin. Control IgG represents normal rabbit IgG (RIgG), which replaced the anti-calcineurin antibody in the precipitation process. (**B**) WAVE1 serine phosphorylation was detected by co-immunoprecipitation. Control IgG represents normal rabbit IgG, which replaced the anti-calcineurin antibody in the precipitation process. (**C**) WAVE1 serine phosphorylation was quantified based on the expression of total WAVE1 in the sample precipitated with the anti-calcineurin antibody. (**D**) Western blot of synaptopodin expression in WAVE1-overexpressing podocytes. (**E**,**F**) WAVE1 and synaptopodin expression levels were quantified after normalization to GAPDH expression. (**G**) Western blot of WAVE1 expression in synaptopodin-knockdown podocytes. (**H,I**) Synaptopodin and WAVE1 expression levels were quantified after normalization to GAPDH expression. The data are presented as the mean ± SD. n = 3. *P < 0.05, **P < 0.01; NS, not significant.

We investigated the involvement of the calcineurin-WAVE1 interaction and WAVE1 phosphorylation in the regulation of podocyte injury by ascertaining whether calcineurin directly interacts with WAVE1 in podocytes. A specific band for WAVE1 was detected after precipitation with the anti-calcineurin antibody (Fig. 6A).

Calcineurin is a serine/threonine phosphatase that regulates substrate dephosphorylation. We further investigated whether calcineurin regulates WAVE1 phosphorylation in podocytes. An immunoprecipitation assay revealed that PAN treatment decreased WAVE1 serine phosphorylation. When the podocytes were pretreated with CsA (0.5 mg/mL) for 1 h before PAN treatment, WAVE1 serine phosphorylation was restored (Fig. 6B,C).

We next overexpressed WAVE1 to further explore the relationship of WAVE1 to synaptopodin. In this experiment, synaptopodin expression was unchanged (Fig. 6D–F). Next, synaptopodin expression was silenced with siRNA; WAVE1 expression was unchanged after synaptopodin knockdown (Fig. 6G–I). These results directly demonstrated that WAVE1 expression does not depend on synaptopodin expression, and vice versa, these results suggest the possibility that CsA regulates WAVE1 in a synaptopodin-independent manner.

### The functions of WAVE1 in podocytes

**Figure 7:**
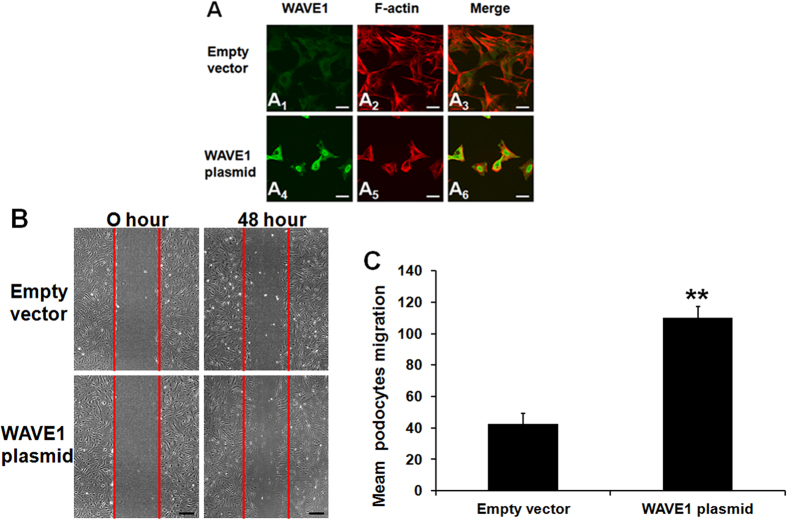
Involvement of WAVE1 in podocyte cytoskeletal structural re-organization and podocyte migration. (**A**) Double-immunolabelling of WAVE1 and F-actin in podocytes transfected with the WAVE1 plasmid. WAVE1 is labelled in green, and F-actin is labelled in red. Scale bar = 20 μm. (**B**) Representative migration results of podocytes transfected with the WAVE1 plasmid. Scale bar = 200 μm. (**C**) Comparison of mean migration effects of podocytes in different groups. The data are presented as the mean ± SD. n = 3. **P < 0.01.

The crucial role of cytoskeletal organization in podocyte function has been widely recognized. We therefore further evaluated the involvement of WAVE1 in podocyte cytoskeletal F-actin structure and cell migration. In WAVE1-overexpressing podocytes, cytoplasmic F-actin reorganized into short, branched actin filaments that were arranged in a disordered manner (Fig. 7A5) compared with empty vector-transfected podocytes, which contained highly ordered, parallel, contractile actin filament bundles (Fig. 7A2). Forty-eight hours after transfection, the migration of WAVE1-overexpressing cells was increased (Fig. 7B,C).

## Discussion

WAVE1, a member of the WASP family, is abundant in the brain and found at low levels in human whole-kidney tissues^18^. Our *in vivo* and *in vitro* experiments demonstrated that WAVE1 was expressed in glomerular podocytes. In particular, IEM on normal rat kidney sections revealed that the majority of WAVE1 labelling was in podocyte cell bodies and FPs.

In our study, we demonstrated that WAVE1 expression increased in both *in vivo* and *in vitro* models of PAN-induced podocyte injury. WAVE1 expression increased with the severity of podocyte injury, suggesting that WAVE1 is involved in podocyte injury. Targeted disruption of the WAVE1 gene in mice elicits central nervous system (CNS)-related effects such as reduced anxiety, sensorimotor retardation, and hippocampal-dependent learning and memory deficits^21^,^22^. WAVE1-knockout mice also exhibit reduced body size and viability. Therefore, WAVE1 is critical for the development and function of the CNS. However, WAVE1 is also a novel target of p35/Cdk5^23^, which inhibits the ability of WAVE1 to activate the Arp2/3 complex *in vitro* and *in vivo*. cAMP signalling reduces WAVE1 phosphorylation, which increases the ability of WAVE1 to activate the Arp2/3 complex. In 2010, Ceglia *et al.*^14^ investigated the molecular mechanisms that mediate neuronal stimulation-induced WAVE1 dephosphorylation in mouse striatal slices and found that calcineurin could dephosphorylate WAVE1 at different sites that were phosphorylated in response to stimulation with various factors.

Whether WAVE1 expression and distribution are regulated by CsA is unclear. Our results showed that CsA treatment restored WAVE1 expression and partially reversed the disordered F-actin arrangement after PAN injury. Furthermore, WAVE1 was regulated by CsA both *in vivo* and *in vitro*.

The above results suggest that WAVE1 may be another substrate of calcineurin. In the present study, although we have not proven that WAVE1 is a direct substrate of calcineurin, the co-immunoprecipitation assay indeed demonstrated that calcineurin directly bound to and interacted with WAVE1 in cultured mouse podocytes. Calcineurin is a serine/threonine phosphatase that regulates substrate dephosphorylation. There have been reports that WAVE is a phosphoprotein^24^,^25^,^26^,^27^,^28^. In 2006, Kim *et al.* reported that the phosphorylation state of WAVE1 at three distinct residues controls its ability to regulate actin polymerization and spine morphology; cyclin-dependent kinase 5 (Cdk5) phosphorylates WAVE1 at Ser310, Ser397 and Ser441 at a high basal stoichiometry, resulting in the inhibition of WAVE1 activity^23^. Calcineurin immunoprecipitation assays were performed to further investigate whether calcineurin regulates WAVE1 phosphorylation in podocytes; these experiments revealed that PAN treatment decreased WAVE1 serine phosphorylation. When podocytes were pretreated with CsA (0.5 mg/mL) for 1 h before PAN exposure, WAVE1 serine phosphorylation was restored.

Synaptopodin is a well-characterized target of CsA. Synaptopodin expression was silenced with siRNA to examine whether CsA regulates WAVE1 in a synaptopodin-independent manner. WAVE1 expression was unchanged by synaptopodin knockdown. Furthermore, synaptopodin expression was unchanged by WAVE1 overexpression. These results directly demonstrated that WAVE1 expression is not dependent on synaptopodin expression, and vice versa, which suggests the possibility that the protective effect of CsA in podocytes via WAVE1 is independent of its effect on synaptopodin.

All WASP/WAVE proteins have a C-terminal verprolin homology/central/acidic (VCA) domain, in which V (WH2) binds monomeric actin and C and A bind the Arp2/3 complex^29^. Through these interactions, WASP/WAVE proteins regulate actin polymerization. Accumulating evidence has demonstrated that cytoskeletal filament rearrangement is the common final event in podocyte FP fusion. WAVE1 overexpression disrupted the F-actin structure compared with the empty vector group, suggesting that WAVE1 is essential for the regulation of cytoskeletal organization. FP fusion is involved in podocyte migration^30^. WAVE1 overexpression promoted podocyte migration. In mouse embryonic fibroblasts, WAVE1 deletion generates a deeper leading-edge actin network, with reduced F-actin staining intensity^31^. In 2015, Sweeneya *et al.* reported a novel role for WAVE1 in controlling the actin network growth rate and architecture. They reported that WAVE1 had dual functions in actin network formation, serving to both stimulate actin nucleation by the Arp2/3 complex and decrease the rate of filament elongation^32^. These reports may help explain why WAVE1 overexpression in podocytes resulted in disorganized, short, and branched F-actin structures. All of these functional experiments suggest that WAVE1 is crucial for maintaining the relatively quiescent and digitated cytoskeletal structure of podocytes.

In conclusion, this study is the first to report that the actin nucleator WAVE1 is expressed in glomerular podocytes and localized in podocyte FPs. Both total and phosphorylated WAVE1 are involved in PAN-induced nephropathy and podocyte injury. The antiproteinuric effects of CsA are mediated by the regulation of WAVE1 phosphorylation. Therefore, WAVE1 may be a novel molecular target for the maintenance of podocyte FPs and for antiproteinuric treatment in the future.

## Methods

### Ethics statement

Study protocols were approved by the Ethics Committee of Peking University First Hospital. All experiments were performed in accordance with approved guidelines of Peking University First Hospital.

### Animals

Male Sprague-Dawley rats (n = 15, 6–8 weeks old) were purchased from the Experimental Animal Center at Peking University Health Science Center. The rats were divided into three groups. The first group (n = 5) was treated with normal saline, the second group (n = 5) received a single intraperitoneal injection of puromycin aminonucleoside (PAN) (150 mg/kg body weight, Sigma-Aldrich, St. Louis, MO, USA), and the third group was treated with CsA (5 mg/kg body weight, Sandimmun; Sandoz Pharma, Basel, Switzerland) daily for 11 days after a single intraperitoneal injection of PAN on the second day. The exact procedure was performed as previously described^13^.

Twenty-four-hour urine was collected on day 10 to measure urinary proteins using a pyrogallol red-molybdate dye-binding method on an automatic biochemical analyser (7170A, Hitachi, Japan). All of the rats were sacrificed on day 10, and the kidneys were removed. One kidney was used to isolate glomeruli using the differential sieving method^33^. The renal cortex of the other kidney was divided into three parts. One part was fixed in 3% glutaraldehyde for transmission electron microscopy, one was embedded in Optimum Cutting Temperature (OCT) compound (Sakura, USA) for immunofluorescence, and one was stored at −80 °C for Western blotting.

Ultrastructural changes in glomerular podocytes were evaluated by transmission electron microscopy. Renal cortex samples that were stored in 3% glutaraldehyde were further fixed in 1% osmium tetroxide, dehydrated in graded ethanol, washed in acetone, and embedded in Epon 812. Ultrathin sections were stained with lead citrate and uranyl acetate and examined on a transmission electron microscope (JEM-1230, JEOL, Japan).

### Cell culture and drug treatment

Primary podocytes were cultured from *Podocin-Cre; Rosa-DTR*^*flox*^ mice, which were kindly provided by the Yale School of Medicine. High-purity primary podocytes were obtained using a previously described method with slight modifications^34^. Briefly, kidneys were cut into small pieces and incubated with 1 mg/ml type I collagenase (Sigma, St. Louis, MO, USA) at 37 °C for 45 min with occasional agitation; then, the cell suspension was filtered through a 40-μm cell strainer (BD Biosciences, Oxford, UK), and the resulting cells were seeded onto multiple pre-coated rat tail collagen plates. Forty-eight hours after seeding, diphtheria toxin-containing medium (Sigma, 100 ng/ml) was applied to the culture for two weeks.

Immortalized mouse podocytes were cultured under growth-permissive conditions on rat tail collagen type I-coated plastic dishes (Corning, Franklin Lakes, NJ, USA) at 33 °C in RPMI 1640 medium (Invitrogen, Carlsbad, CA, USA) supplemented with 10% foetal bovine serum (Gibco BRL, Gaithersburg, MD, USA), 10 U/mL mouse recombinant γ-interferon (Sigma), and a 100 U/mL penicillin/0.1 mg/mL streptomycin mixture (Gibco). Podocytes were grown in a flask at 37 °C with 5% CO_2_ for a minimum of 10–14 days to allow for differentiation. Cells were then plated in 6-well plates for Western blotting and in plates with glass coverslips for immunofluorescence staining. All experiments were performed at 70% confluence. Podocytes were pretreated with CsA (0.5 mg/mL) for 1 h before exposure to PAN for 24 h.

### Conventional reverse transcription polymerase chain reaction (RT–PCR)

Conventional RT–PCR was performed in primary cultured podocytes. Total RNA was isolated using TRIzol reagent (Invitrogen). Two micrograms of RNA was reverse transcribed using a High Capacity cDNA Reverse Transcriptase Kit (Invitrogen). WAVE1 PCR was performed for 35 cycles, and the PCR products were observed by agarose gel electrophoresis. The amplification of WAVE1 in mouse cerebral cortex tissue served as a positive control.

### Quantitative real-time PCR

Total RNA was isolated from cultured podocytes using TRIzol reagent (Invitrogen). Two micrograms of RNA was reverse transcribed using a High Capacity cDNA Reverse Transcriptase Kit (Invitrogen) according to the manufacturer’s protocol. The following primers were used for quantitative real-time PCR: GAPDH cDNA, 5′-CGGAGTCAACGGATTTGGTCGTAT-3′ (sense) and 5′-AGCCTTCTCCATGGTGGTGAAGAC-3’ (antisense); and WAVE1 cDNA, 5′-GGGAACGAGCCTTATTCCGT-3′ (sense) and 5′-AGTGGGAAGGGGTTCTGCTA-3′ (antisense). Quantitative real-time PCR was performed using a SYBR Green PCR Master Mix Kit (Invitrogen). The cycling conditions included denaturation at 95 °C for 10 min followed by 35 cycles of annealing at 95 °C for 15 s and extension at 64 °C for 1 min. The relative mRNA expression was normalized to GAPDH expression and calculated using the delta-delta method from the threshold cycle numbers. Based on the exponential amplification of both the target gene and the calibrator, the relative expression of a target gene at a particular threshold cycle is represented by 2^−∆∆Ct^.

### Western blotting

Podocytes were lysed with RIPA buffer containing a protease inhibitor cocktail (Roche, USA). Fifty micrograms of total protein was subjected to 8%–15% SDS-PAGE and transferred to nitrocellulose membranes (Amersham Biosciences, Piscataway, NJ, USA). After the blots were blocked with PBS containing 5% non-fat dry milk for 1 h at room temperature, they were incubated overnight (14–16 h) at 4 °C with the following primary antibodies: rabbit anti-WAVE1 (1:1,000; Abcam, UK), mouse anti-synaptopodin (1:1,000; PROGEN, Germany), rabbit anti-phospho-serine (1:500; Rockland, USA), rabbit anti-nephrin (1:1,000; Sigma, USA), and mouse anti-GAPDH (1:5,000; Chemicon, Temecula, CA, USA). Subsequently, the membranes were rinsed three times for 5 min each in PBS buffer with 0.05% Tween-20 and incubated with horseradish peroxidase-conjugated anti-rabbit or anti-mouse IgG (Santa Cruz Biotechnology, Santa Cruz, CA, USA). After a final wash, the membranes were developed using an enhanced chemiluminescence reagent (Millipore, Boston, MA, USA), and the specific protein bands were scanned and quantitated relative to GAPDH. The densitometric analysis of the images was performed using Image J software (National Institute of Mental Health, Bethesda, MD, USA).

### Co-immunoprecipitation assay

Co-immunoprecipitation experiments were conducted according to the manufacturer’s instructions (22202, Beaver, China). Total protein from the cultured podocytes was extracted using lysis buffer (1.0% Triton X-100, 150 mM NaCl, 5 mM EDTA, 1 mM PMSF, and 20 mM Tris, pH 7.5) that contained a protease inhibitor cocktail (P8340, Roche, USA). The samples were centrifuged at 13,000 g for 10 min at 4 °C. A calcineurin rabbit polyclonal antibody (2 μg/500 μg total protein; Cell Signaling Technology, USA) was added to the supernatant, and the mixture was rotated overnight at 4 °C. Then, the mixture was loaded with protein A+G agarose and incubated for 30 min at room temperature. The centrifuged sediment was retained and mixed with 1× loading sample buffer. The samples were boiled at 70 °C for 10 min and were then analysed by Western blotting. Normal non-immune rabbit antibody (Santa Cruz) was used as a control.

### Small interfering RNA (siRNA) experiment

Synthetic siRNA targeting mouse synaptopodin and non-targeting control siRNA were obtained based on previously reported sequences^13^. High-knockdown-efficiency siRNA was used. Transfections were performed with Lipofectamine RNAi MAX reagent (Invitrogen) according to the manufacturer’s instructions. Forty-eight hours after transfection, total protein extracts from the transfected cells were used for Western blot analysis.

### WAVE1 overexpression experiment

The plasmid encoding mouse WAVE1, pCMV6-Kan/Neo-WAVE1, and the corresponding empty vector, pCMV6-Kan/Neo, were purchased from Origene (Origene, Rockville, MD, USA). Podocytes were transiently transfected with pCMV6-Kan/Neo-WAVE1 or pCMV6-Kan/Neo using Lipofectamine 2000 transfection reagent (Invitrogen) according to the manufacturer’s instructions. Forty-eight hours after transfection, the transfection efficiency was determined by Western blotting.

### Fluorescence confocal microscopy

Five-micrometre cryosections were fixed in ice-cold acetone, permeabilized with 0.3% Triton X-100 and blocked with 10% goat serum. The following primary antibodies were used: rabbit anti-WAVE1 (1:200; Abcam, UK) and mouse anti-synaptopodin (1:100; PROGEN, USA). After three washes, the slides were incubated with Alexa Fluor® 488 goat anti-rabbit IgG (1:200; Invitrogen) and Alexa Fluor® 596 goat anti-mouse IgG (1:200; Invitrogen). Cells on coverslips were fixed with 4% paraformaldehyde, permeabilized with 0.3% Triton X-100 and blocked with 10% goat serum. Primary and secondary antibodies as well as Alexa-phalloidin (1:200; Invitrogen) were applied. The slides were mounted with 50% glycerol. Images for each antibody at the same light exposure were obtained by confocal laser-scanning microscopy (TCS-SP5, Leica, Germany). Photographs of WAVE1-stained glomeruli and podocytes were selected randomly and analysed by a person who was blinded to the study groups.

### Immunoelectron microscopy (IEM)

Samples from normal rat renal cortices were fixed in 3% paraformaldehyde in 0.1 M phosphate buffer. The monoclonal antibody against WAVE1 was used at a concentration of 1:50. The secondary antibody was conjugated to 5-nm gold particles. The sections were examined as previously described^35^. The IEM analyses of the WAVE1 signal were conducted in a blinded fashion.

### Cell migration assays

For the migration assays, wounds were made with a sterile pipet tip after the cells had grown to complete confluence in a 6-well plate. Images were acquired on an inverted phase-contrast microscope just before (0 h) and 48 h after wound formation. The number of cells crossing the wound border was calculated to estimate cell migration.

### Statistical Analysis

All of the experiments were performed at least three times, with similar results obtained between experiments. All the data are presented as the mean ± SD. Statistically significant differences were assessed using Student’s t-test or one-way analysis of variance (ANOVA) for multiple comparisons. Statistical significance was set at a P value of <0.05.

## Additional Information

**How to cite this article**: Li, X. *et al.* Cyclosporine A protects podocytes by regulating WAVE1 phosphorylation. *Sci. Rep.* **5**, 17694; doi: 10.1038/srep17694 (2015).
